# Isolated Sixth Nerve Palsy: A Case of Pseudotumor Cerebri and an Overview of the Evolutionary Dynamic Geometry of Dorello's Canal

**DOI:** 10.7759/cureus.15340

**Published:** 2021-05-30

**Authors:** Hassan Kesserwani

**Affiliations:** 1 Neurology, Flowers Medical Group, Dothan, USA

**Keywords:** sixth cranial nerve palsy, benign intracranial hypertension, ophthalmology, severe headache, headache in children

## Abstract

The dynamics of increased intracranial pressure (ICP) and sixth cranial nerve palsy has undergone a paradigm shift, with emphasis shifting from a length hypothesis to a theory based on novel anatomic findings pertaining to the geometry of Dorello's canal. In particular, the sixth cranial nerve resides in a transfixed coaxial cylinder within the canal. The cisternal portion of the nerve is intradural and the rest of the nerve is extradural; therefore, with increased ICP, the former is stretched, thereby pulling on the rest of the nerve, which is anchored in Dorello's canal. We present a case of pseudotumor cerebri secondary to minocycline presenting with an isolated sixth nerve palsy. This case is used as a platform to segue into the recent findings outlined above, in particular, the evolutionary transformation of Dorello's canal from a circular outline with a bony roof to an elliptic profile with a fibro-osseus roof during hominid basocranial expansion. The fibro-osseus roof, being elastic, is particularly susceptible to the influence of raised ICP, thereby narrowing the canal and injuring the sixth cranial nerve.

## Introduction

The sixth cranial nerve is quite unique: a pure motor nerve, the second longest cranial nerve after the trochlear nerve, entirely dedicated to supplying the lateral rectus muscle, thereby allowing abduction of the eye. Its course at the base of the brain is remarkable - after leaving the ponto-medullary junction, it enters the subarachnoid space (the cisternal segment), and after a variable distance, it penetrates the dura (intradural segment), leaves the intramural space, and travels through the osteofibrous Dorello's canal under the petrosphenoidal ligament (PSL), also known as Gruber's ligament, stretching between the petrous apex (spine) and the accessory clinoid process (ACP) [1]. While here it is anchored by a meningeal tube - a rigid immobile cylinder within Dorello's canal - as a co-axial cylinder [2]. It then climbs up the wall of the clivus and penetrates the cavernous sinus, riding along with the inferior pertrosal sinus. In the cavernous sinus, it lies immediately lateral to the pericarotid sympathetics. After leaving the cavernous sinus, it makes a sharp, almost ninety-degree turn upwards and forwards and then enters the superior orbital fissure at the orbital apex surrounded by the annulus of Zinn. The latter is a fibrous ring formed by the tendons of the four recti muscle. Through it also enter cranial nerves III, IV, the ophthalmic division of V, ophthalmic vein, and sympathetic fibers. The optic nerve, ophthalmic artery, and central retinal vein pass through the optic canal [3].

During this treacherous journey, the sixth cranial nerve is highly vulnerable to injury, especially with high intracranial pressure. This was thought to be due to its long intracranial course, however, a study of 26 cadavers showed that the sixth cranial nerve is one-third the length of the trochlear nerve, and the former is more likely to be injured by raised ICP [4]. The sixth cranial nerve is tethered at two points: at its exit from the pontomedullary junction and at Dorello's canal. As the pontomedullary junction descends during transtentorial herniation due to raised ICP, the sixth nerve is stretched like a bow-string and pulled at Dorello's canal, leading to ischemic necrosis. In the pontine cistern, the sixth cranial nerve is traversed and indented by branches of the basilar artery, especially the anterior inferior cerebellar artery (AICA), and posterior fossa tumors may distort the anatomy and lead to a sixth nerve palsy by a pressure-mass effect. The parallel course of the sixth cranial nerve along the clivus may lead to a palsy from the mass effect of a chordoma [5]. With the cavernous sinus syndrome from a malignancy such as a meningioma, carotid siphon aneurysm or fistula, fungal infection, vasculitis or the Tolosa-Hunt syndrome, a sixth nerve palsy may arise from direct pressure or inflammation. The close proximity of the sixth cranial nerve to the peri-carotid sympathetics may lead to a Horner's syndrome, the combination of a sixth nerve palsy and a Horner syndrome being virtually pathognomic of a cavernous sinus syndrome [6,7].

The differential diagnosis of an isolated sixth nerve palsy includes ocular myasthenia gravis, thyroid ophthalmopathy, and microvascular disease from diabetes and hypertension. For the sake of completion, we will mention the brainstem syndromes of Raymond, Millard-Gubler, and Foville syndrome as they are not etiologies of isolated sixth nerve palsy and also involve other lower cranial nerves, gaze centers, and long tracts. Gradenigo's syndrome with petrous apex spread from otitis media via the inferior petrosal sinus involves cranial nerves V, VI, VII, and VIII, and is of historical interest [8].

Lastly, as in our case report, a sixth nerve palsy can be an isolated finding in patients with pseudotumor cerebri/idiopathic intracranial hypertension and can be seen in up to 30% of cases. The injury is likely due to stretching of the sixth cranial nerve but is almost always reversible and therefore a neuropraxia [9].

## Case presentation

A 17-year-old young woman presented with an insidious onset of unremitting severe bilateral occipital headaches associated with a roaring sound in her left ear. The severity of the headache required her to seek care in the emergency room twice. One week into her illness, a fixed horizontal diplopia with maximal separation of images with left direction of gaze had developed, necessitating the use of an eye patch. She did not report any transient visual obscurations or other vision loss. Despite being overweight, no increase in weight was recorded over the previous year. Her past medical history was significant for acne, for which was treated with monocyline 100 milligrams (mg) daily for the last three months. Otherwise she was a healthy young woman.

**Figure 1:**
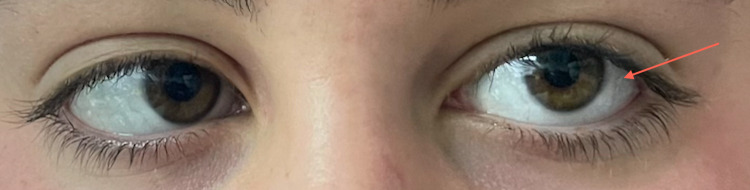
Failure of abduction of the left eye consistent with a left sixth nerve palsy (red arrow).

On examination, her blood pressure is 112/68 mmHG, with a pulse of 98 beats per minute. Her height is 5 feet and 5 inches with a weight of 241 pounds and a body mass index of 40.1 kg/m^2^. Gait cadence, tandem-walking, and heel-and-toe walking were normal. Ocular motion reveals a frank left sixth nerve palsy (Figure 1).

**Figure 2:**
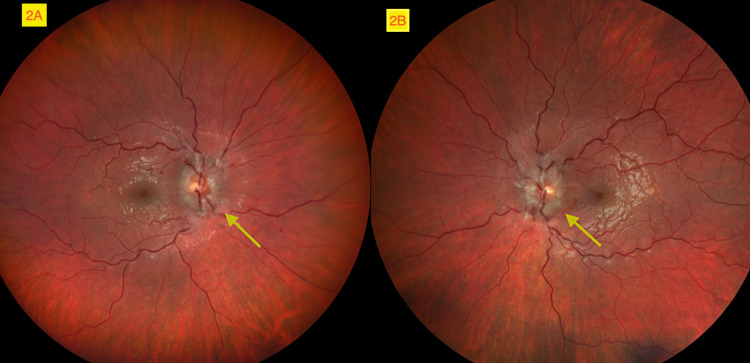
Bilateral optic disc edema with blurring of optic disc margins and reduction of blood vessels (yellow arrows). 2A: left eye, 2B: right eye.

No Horner's syndrome or Marcus-Gunn pupil was noted. Consensual and accommodative reflexes were normal. Funduscopic examination with pupil dilation was performed by an ophthalmologist and revealed bilateral papilledema (Figure 2).

Facial sensation and power were symmetric and preserved. The rest of the cranial nerve examination was normal. Power testing with the medical research council (MRC) scale was 5/5 throughout both upper and lower limbs. Deep tendon reflexes were lively in upper and lower limbs. Touch-pressure, vibratory, and joint position sense were normal in the fingers and toes.

**Figure 3:**
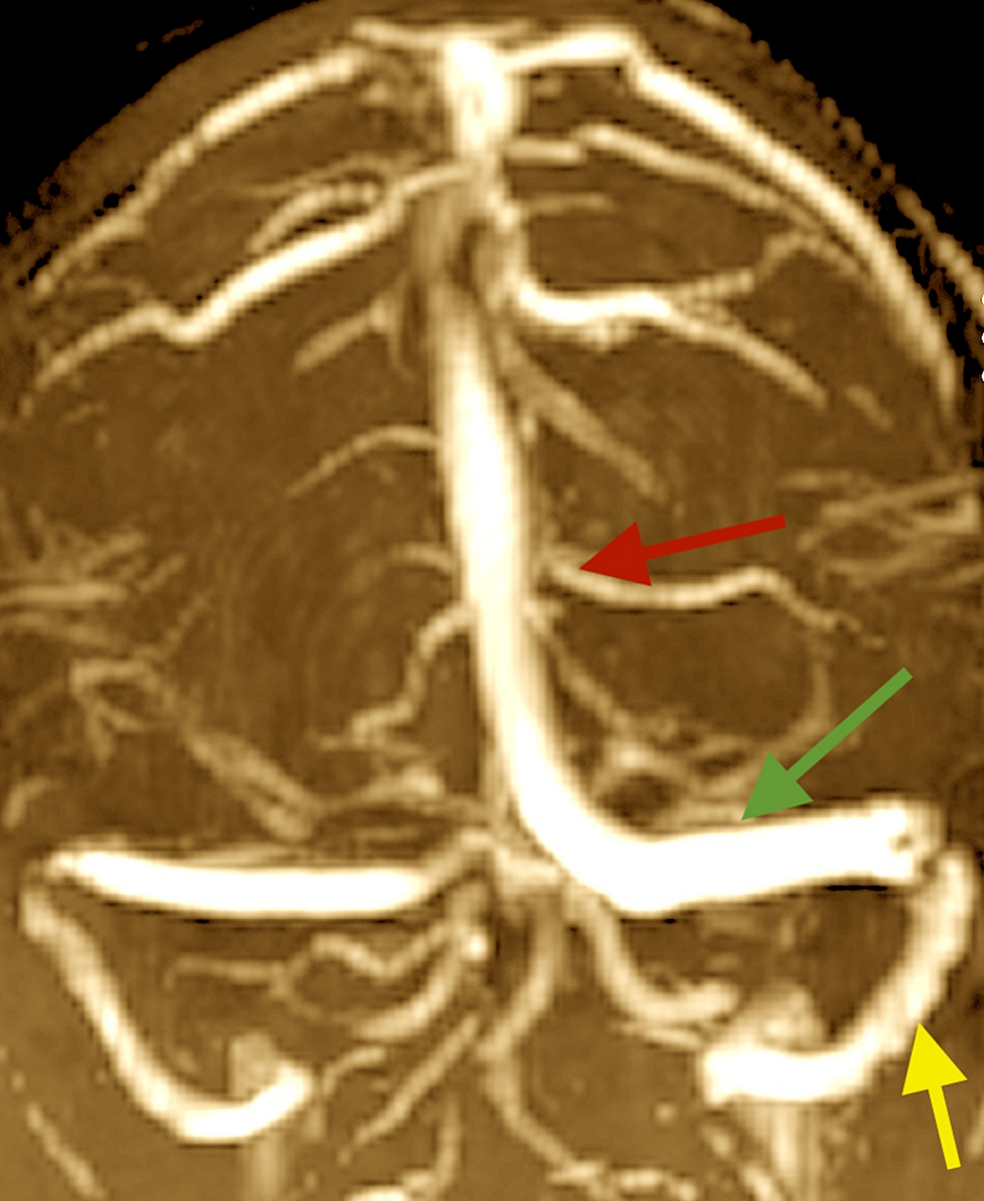
Magnetic resonance venography (coronal section) showing patent major venous sinuses bilaterally. Left superior sagittal sinus (red arrow), transverse sinus (green arrow) and sigmoid sinus (yellow arrow). MRV: magnetic resonance venography

A magnetic resonance imaging (MRI) of the brain was normal with and without contrast. A magnetic resonance venogram (MRV) showed patent venous sinuses (Figure 3).

**Figure 4:**
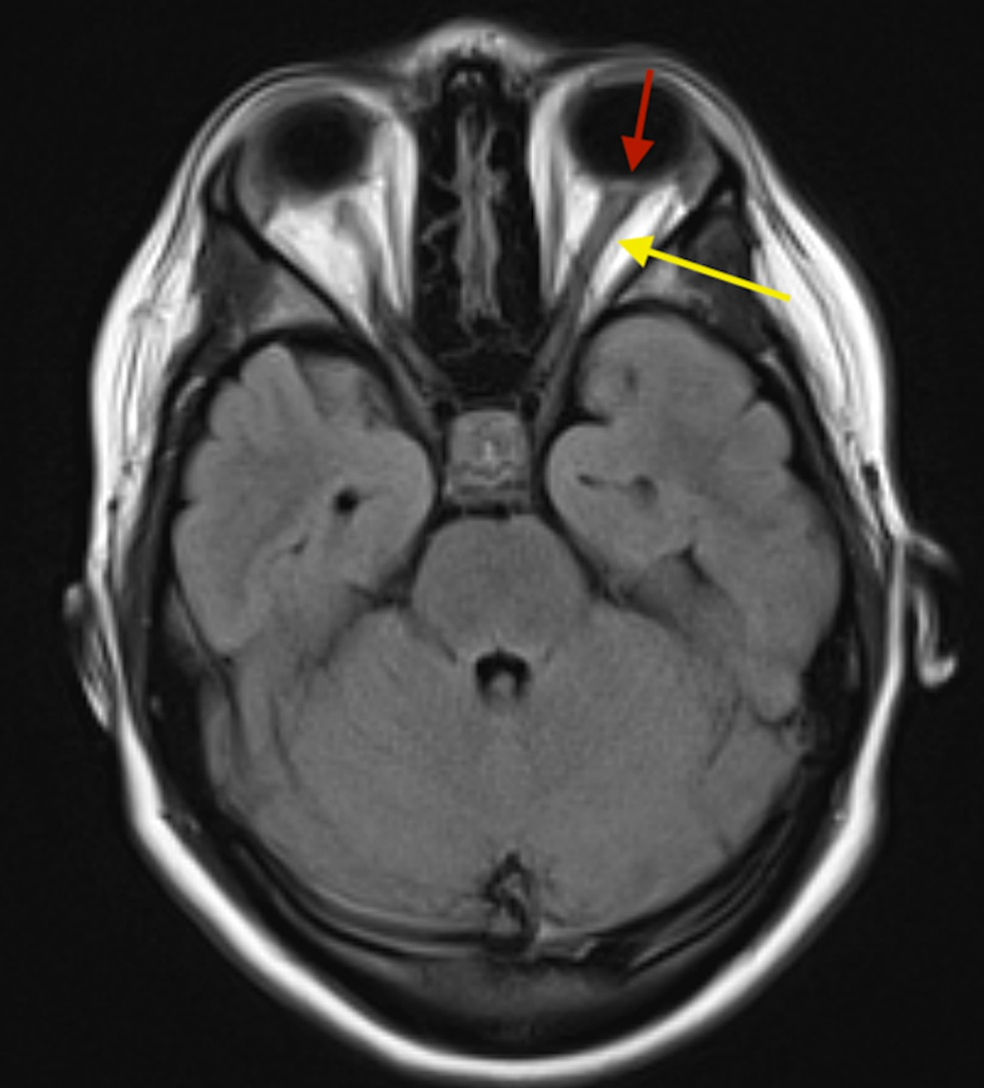
FLAIR MRI of the brain (axial section) showing thickened optic nerve sheath (yellow arrow) and flattened lamina cribrosa of sclera (red arrow). FLAIR MRI: Fluid-attenuated inversion recovery magnetic resonance imaging

An MRI of the brain and orbits with and without contrast showed bilateral optic nerve sheath distention with flattening of the posterior bulge of the sclera (lamina cribrosa) consistent with optic disc edema (Figure 4).

A supine lumbar puncture in the lateral decubitus position revealed a high cerebrospinal fluid (CSF) pressure, greater than 55 centimeter (cm) of water pressure (normal: less than 25 cm of water). CSF revealed normal protein and no pleocytosis. The patient was diagnosed with pseudotumor cerebri secondary to minocyline therapy, a well-established and tight association. Minocycline was discontinued and the patient was started on a slowly escalating dose of 250 mg of acetazolamide daily with a target dose of 750 mg daily, as tolerated. By the end of the first week, the headache had completely resolved, and by the end of the second week, the diplopia had resolved. At the two-month ophthalmologic visit, the optic disc edema had also resolved and the patient was weaned off acetazolamide.

## Discussion

**Figure 5:**
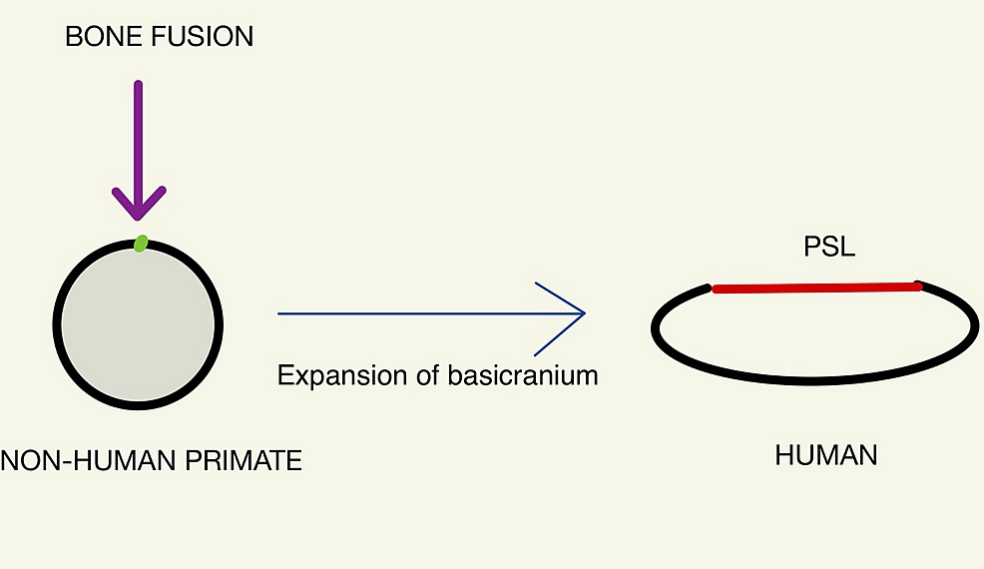
Expansion of the basicranium and conversion of a circular Dorello's canal to an elliptic canal in humans with replacement of a bony roof (violet arrow) to a fiber-ligamentous roof (red line), PSL, as we transition from a non-hominid primate to a hominid primate. PSL: petrosphenoid ligament

Dorello's canal, also known as the petroclival segment, marks the transition between the posterior and middle cranial fossa. This canal has undergone evolutionary change being fused superiorly by a bony arch in non-hominid primates, the union of the petrous apex (spine) and the ACP. With hominid brain expansion and growth of the basicranium, the non-union of the petrous apex and ACP was replaced by an elastic fibro-osseus ligament, the PSL, covering the roof of the foramen. The canal is elliptic in shape in humans and rounded in non-human primates, the expansion of the cranial base converting it from a circle to an ellipse (Figure 5) [10].

The elastic nature of the PSL allows high ICP to squeeze and compress the PSL and constrict the sixth cranial nerve in Dorello's canal leading to a sixth nerve palsy. Furthermore, the PSL is butterfly-shaped, with a narrowing or stricture in the middle in 78% of cases. Meanwhile, the sixth cranial nerve runs in the middle of the canal, where it is susceptible to compression by the vertical bending forces on the middle portion of the elastic PSL into Dorello's canal [11]. In non-hominid primates, this is not possible as the roof of Dorello's canal is bony.

The cisternal segment of the sixth cranial nerve is intradural and therefore subject to raised ICP. Meanwhile the rest of the sixth cranial nerve, including the petroclival and cavernous segments, are extradural and not subject to raised ICP. Therefore, with a diffuse and isotropic increase in ICP, the nerve is subject to a differential pressure with the proximal cisternal segment of the nerve pulling or tugging on the rest of the nerve; this being a tentative explanation of a neuropraxia of the nerve. Contrast this to transtentorial herniation where there is global stretch of the nerve with descent of the pontomedullary junction and massive increases in ICP (plateau waves) and subsequent necrosis of the nerve due to stretching of the nerve beyond its yield point (Hooke's law) [12,13].

**Figure 6:**
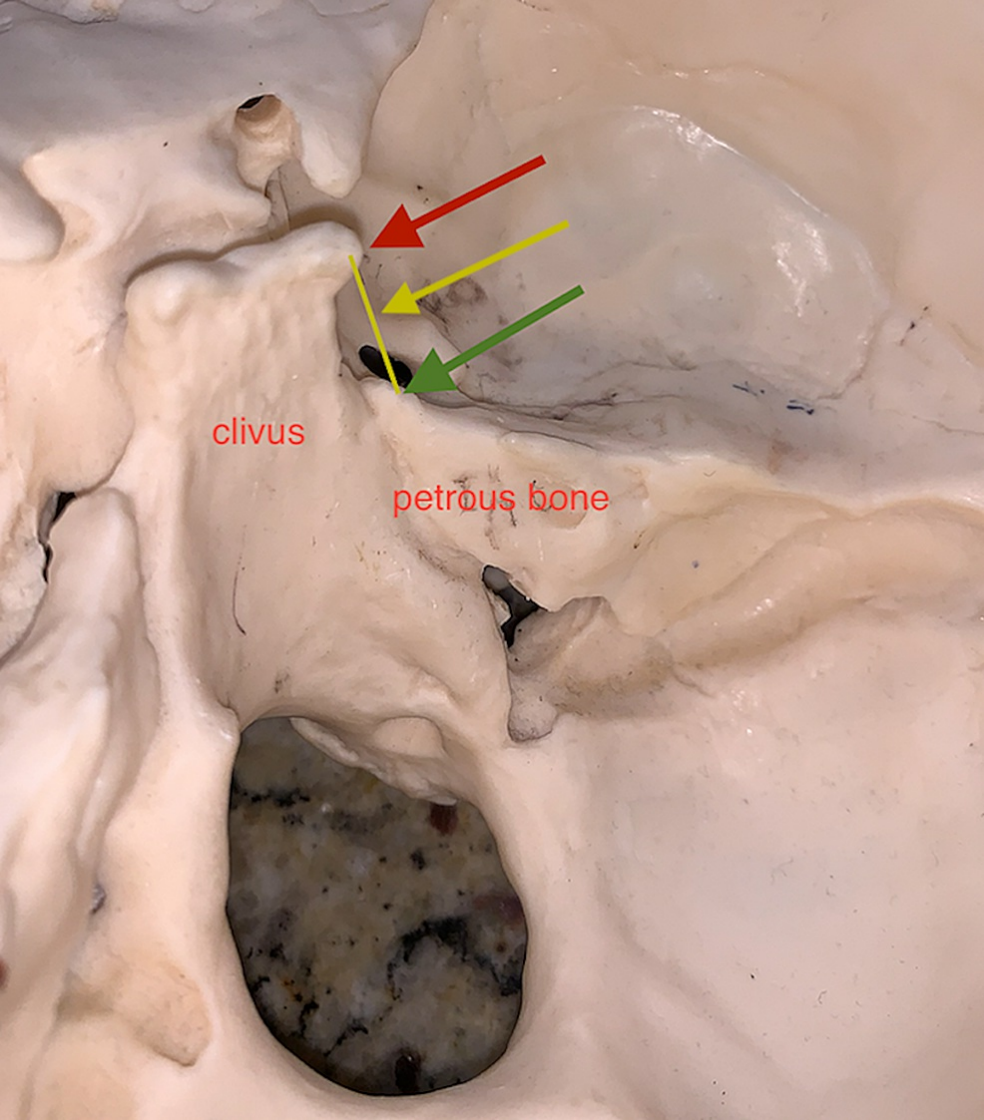
Oblique view of right Dorello's canal - posterior clinoid process (red arrow), petrous spine (green arrow), petro-clinoid ligament (yellow arrow).

With Dorello's canal being defined as the space or trough between the clivus and petrous bone bounded superiorly by the the PSL, anatomic variations do occur and this space is often referred to as the petroclival region (Figure 6).

There are instances wherein the sixth cranial nerve courses over Dorello's canal and some authors have suggested an alternative appellation; the petroclival venous confluence (PVC). This region is akin to chamber within the confines of the meningeal and periosteal layers of the dura with the confluence of multiple venous sinuses draining into the inferior petrosal sinus [14]. The PVC is divided into two compartments: an upper compartment carries the sixth cranial nerve and the lower compartment, the inferior petrosal sinus. This is similar to the situation seen with the jugular foramen, which is divided into two compartments: the pars venosa (internal jugular vein and cranial nerves X, XI, and Arnold's nerve) and pars nervosa (cranial nerve IX, and Jacobsen's nerve) [15].

## Conclusions

Evolutionary developmental biology or "evo-devo" is an exciting field that helps explain functional anatomy pertaining to evolutionary descent. The expansion of the basicranium parallels the growth of the brain during hominid evolution. The morphological changes are many, one of which pertains to a classic and frequent presentation in neurological practice: a sixth nerve palsy. Localization of a sixth nerve palsy has a well-established differential diagnosis and is an exercise of neurological finesse and beauty. However, classical neurologists have also noted the "false localizing sign" of a sixth nerve palsy in cases of diffuse and isotropic increase in ICP. Here there is no focal disease but a global process, which as a quirk of neuroantomy has predisposed this nerve to compression at Dorello's canal due to the transformation of the roof from an osseus structure to a fibro-elastic and compressible tent as a result of brain expansion during hominid evolution. This is the classic "false localizing sign" of a sixth cranial nerve palsy, which now has a plausible and beautiful explanation.
